# Pastors as Partners in Care: African Immigrant Pastors’ on Mental Health Care Referral Processes for Young Congregants Experiencing Symptoms of Psychosis in the US

**DOI:** 10.1007/s10597-024-01335-x

**Published:** 2024-08-20

**Authors:** Neely Myers, Robert Meeker, Valerie Odeng

**Affiliations:** 1 Department of Anthropology, Southern Methodist University, Dallas, TX USA; 2 Department of Psychiatry, University of Texas – Southwestern Medical School, Dallas, TX USA; 3 Crown Family School of Social Work, University of Chicago, Chicago, IL USA; 4 The George Washington University – Milken Institute School of Public Health, Washington, DC USA

**Keywords:** African immigrants, Mental illness, Mental health, Help-Seeking, Referral, Faith-based

## Abstract

Early support for young people experiencing psychosis is key to preventing negative outcomes. First and second-generation Black immigrants to predominantly white countries are at higher risk for psychosis (Bourque et al. in Psychol Med 41(5):897-910, 2011) and novel interventions are needed to help support immigrants youths and families. African immigrant pastors are culturally valued and poised to help congregants with psychosis and their families, but we know little about the supports pastors offer and what kinds of tools they might need to address the needs of their congregants. This qualitative study explores semi-structured interviews with 16 primarily nondenominational, Christian, African immigrant pastors to elucidate how they served young adult congregants experiencing symptoms of psychosis and their families. Using grounded theory analytic methods, five key themes emerged: (1) building supportive relationships; (2) identifying the source; (3) healing the problem; (4) families as partners in care; and, (5) referring congregants to and collaborating with mental health professionals. These findings describe an initial set of care practices as a starting point for understanding the current and future role of African immigrant pastors as partners in providing mental health care.

First- and second-generation Black immigrants to predominantly white countries are at higher risk for developing psychosis (Bourque, Ven & Malla, 2011). Psychosis is described as a “complete break with reality,” which can include hearing voices others cannot hear or seeing things others cannot see, or delusions such as that one is a famous religious or political figure (Compton & Broussard, 2009). Psychosis symptoms can occur across multiple psychiatric disorders, including PTSD, depression, bipolar disorder and schizophrenia.

Providing appropriate and adequate mental health supports for Black African immigrants to the U.S. is important (Akinsulure-Smith, 2017; Venters et al., 2010). For African descendants, increased time spent in the U.S. has been associated with poorer mental health status than other racial and ethnic groups (Akinsulure-Smith, 2017; Gee et al., 2006). Adverse conditions faced before or during transnational immigration, social deprivation, separation from parents, and perceived and real discrimination upon arrival increases psychosis risk (Cantor-Graae & Selten, 2005; Hjern et al., 2004; Wicks et al., 2005). Moreover, all Black persons in the U.S. suffer disproportionately from structural racism-related social and environmental conditions that increase psychosis risk (Anglin et al., 2021).

African immigrants who are at increased risk of developing psychosis can thus benefit from reliable, early mental health supports. Early intervention can prevent adverse outcomes and promote recovery (Azrin et al., 2015). Early referrals to specialized care for young people with psychosis can prevent negative outcomes like interpersonal violence, homelessness, and suicide (Marshall et al., 2005; Perkins et al., 2005).

However, help-seeking delays for Black immigrants are common, making it harder for them to access early care. Barriers include stigma concerns, treatment costs, a lack of information about available services, and a lack of language interpreters or substantive efforts to provide culturally attuned care (Akinsulure-Smith, 2017; Boise et al., 2013; Ferrari et al., 2015; Nadeem et al., 2007; Pederson et al., 2021; Saechao et al., 2012). There is a limited literature on how to best address the needs of this community even though they are at an elevated risk for psychosis.

One underexplored mental health resource for African immigrants is religion and spirituality.^1^ Many persons ascribe religious and spiritual explanations to experiences of psychosis symptoms like hallucinations and delusions and turn to religious professionals initially for help (Bhikha et al., 2012; Jones et al., 2016; Larøi et al., 2014; McCarthy-Jones et al., 2013; Napo et al., 2012). In addition, religious or spiritual healing for mental health concerns often appeals to African immigrants (Adekeye et al., 2014; Boise et al., 2013; Filippi et al., 2014; Nadeem et al., 2008, p. 9). 82% of African and Caribbean Black immigrants in one study viewed religious faith as helpful for dealing with an emotional problem, compared with 19% who endorsed medication as helpful (Nadeem et al., 2008). In another study, the most frequently used mental health resource among Black African immigrants was spiritual healing: 22% of Ethiopian immigrants and 23% of Nigerian immigrants endorsed it (Chaumba, 2011). One study also suggests religion and spirituality are essential for the treatment of substance use problems for West African immigrants (Senreich & Olusesi, 2016). Others found that West African immigrants (Thomas, 2008) and Nigerian-born immigrant women (Ezeobele et al., 2010) preferred pastoral support for emotional problems. Clergy have also played a significant emotional support role for Caribbean Black immigrants (Chatters et al., 2011).

Unfortunately, despite the heightened psychosis risk and the potential excellence of fit between faith-based care and mental health concerns for Black immigrants, there is little literature on mental health literacy and referral practices among African-born pastors in the US who are likely to interact with African immigrants. In one brief article, three African-born pastors discussed the importance of spirituality for healing (Adu-Boahene et al., 2017). Two other studies discussed collaborations with African immigrant pastors. Adekeye et al. (2014) found concerns with the difficulty of navigating the health care system and challenges due to assimilation pressures. For example, while religious activity was associated by participants with good mental health, being isolated from one’s culture and the need to appear integrated into American culture presented barriers to religious attendance (Adekeye et al., 2014). Oppenheim et al. (2019) also discussed promoting mental health education and awareness in Black immigrant groups through participatory research.

Building on the call for further research on the effectiveness of collaboration and referral processes between religious leaders and mental health services to address the mental health needs of Black immigrants (Derr, 2016, p. 271; Nadeem et al., 2008, p. 9), the present study provides novel insight into the ways 16 African immigrant pastors in North Texas, an area with many African immigrants, responded to a prompt about congregants displaying potential symptoms of psychosis [see vignette, Table 1]. We asked how they supported persons with symptoms of psychosis in their congregations. What emerged was a culturally relevant set of care practices that demonstrated a sophisticated understanding of the delicate balance between religious or spiritual and medical crises, a holistic way of approaching a mental health problem in partnership with families and local medical professionals, and information about what further resources may be helpful for pastors to provide even better support.

## Methods

This study was conducted in North Texas under the supervision of the Southern Methodist University Human Subjects Research Institutional Review Board. The third author, Valerie Odeng, an African immigrant and Masters student in Public Health, identified several other African immigrant-serving pastors who fit our criteria using “snowball sampling” (Barnard, 2018), a strategy where a person who is interviewed for the study is asked to recommend other potential participants. Odeng recruited by telephone and text message, and then obtained signed consent before the interview.

The interviewer first presented the vignette about a young adult with a psychotic disorder (Table 1), which is common in research with non-clinicians, including clergy, to help them understand symptoms of psychosis (Adu et al., 2021; James et al., 2014). Next, the pastors were prompted to share their experience working with Black youth (ages 15–30) in their congregation with mental health concerns. The interviewer asked them to think about how families and youth sought help for such concerns, the pastor’s role and the church’s role in helping people, how and when they directed people to other sources of care, and what further support church communities and families needed.

Audio-recorded interviews were professionally transcribed verbatim, proofread to validate the transcription accuracy, and uploaded into a qualitative data analysis platform, Dedoose. Using grounded theory methods (Charmaz, 2006), the authors jointly developed open codes based on iterative patterns that emerged from initial readings of nine transcripts (Guest et al., 2006). All interviews were then coded by Odeng and Meeker to ensure consistency in application and any issues or discrepancies or questions were discussed in regular team meetings with Myers. Codes were then grouped into overarching “themes” based on conversations among team members about consistent thematic patterns across the data set.

## Results

Twenty-three African immigrant pastors agreed to participate and seventeen completed the study. (See Table 2. Demographics.) One recording file was corrupted. We thus analyzed the remaining sixteen in-depth interviews with primarily male (*n* = 13, 81%) pastors of primarily nondenominational (*n* = 13, 81%), Christian (*n* = 16, 100%) congregations. All reported having a graduate-level education from either the United States (*n* = 8, 50%), Africa (*n* = 5, 32%), both (*n* = 1, 6%), or another region (*n* = 2, 12%). Nearly all had received pastoral education, half (*n* = 8) in the United States. One-fourth (*n* = 4) reported formal training in mental health.

**Table 2:** Pastor demographics (*n* = 16)

Category	*N* (%)
**Gender**	
*Male*	13 (81.0)
*Female*	3 (19.0)
**Age**	
*35–49*	12 (75.0)
*50–65*	4 (25.0)
**Country of Origin**	
Cameroon	7 (4.4)
Ghana	2 (12.5)
Ivory Coast	2 (12.5)
Liberia	2 (12.5)
Nigeria	2 (12.5)
Sierra Leone	1 (6.3)
**Religious Affiliation**	
Nondenominational	13 (81.0)
Apostolic	2 (12.5)
Baptist	1 (6.3)
**Education Level**	
Doctoral	6 (37.5)
Master’s	6 (37.5)
Associate’s	1 (6.3)
Not Specified	3 (18.8)
**Location of Education**	
United States	8 (50.0)
Africa	5 (32.0)
United States and Africa	1 (6.0)
Other	2 (12.0)
**Form of Pastoral Education**	
Theology	5 (32.0)
Ministry	1 (6.0)
Pastoral Leadership	1 (6.0)
Christian Apologetics	1 (6.0)
Ontology	1 (6.0)
Divinity	1 (6.0)
Other	2 (12.0)
Unknown	3 (18.0)
**Location of Pastoral Education**	
United States Only	8 (50.0)
Africa Only	2 (12.0)
United States and Africa	5 (32.0)
Other	1 (6.0)
**Formal Mental Health Training**	
Yes	4 (24.0)
No	10 (63.0)
Unknown	2 (12.5)
Church Size*	
Small	4 (25.0)
Medium	8 (50.0)
Large	3 (18.0)
Unknown	1 (7.0)

* Using November 27, 2020 USA Churches (http://www.usachurches.org/church-sizes.htm*)* definitions of church size

**Table 1:** Original Psychosis Vignette and Modifications for Cultural Relevance

Original Vignette	Modified Vignette
John married Mary approximately three years ago, after a two-year courtship. Mary was a devout Catholic, while John came from a background that de-emphasized religious interests. Marriage to Mary was dependent on conversion to Roman Catholicism, and John found himself becoming increasingly interested and involved with his religious training. He went to church regularly, and began to study religious literature and to read the Bible. Over time this religious interest became more and more intense and finally led to a significant commitment of time and effort to the question of dating the birth of Christ. About two years after his marriage, John began to place the birth of Christ later and later in time. Within another six months, he began to suspect that he himself was Christ. At this point his parents and wife sought hospitalization for the patient.	John is a 25-year-old male businessman. John married Mary approximately three years ago, after a two-year courtship. Mary was a devout Catholic, while John came from a background that de-emphasized religious interests. Marriage to Mary was dependent on conversion to Roman Catholicism, and John found himself becoming increasingly interested and involved with his religious training. He went to church regularly and began to study religious literature and to read the Bible. Over time this religious interest became more and more intense and finally led to a significant commitment of time and effort to the question of dating the birth of Christ. About two years after his marriage, John began to place the birth of Christ later and later in time. Within another six months, he began to suspect that he himself was Christ. At this point his parents and wife sought help for him.

Five key themes emerged from these interviews: (1) the importance of building supportive relationships; (2) how to identify the source of the problem as spiritual or medical; (3) how to heal the problem; (4) making families partners in care; and, (5) the importance of referrals to and collaborations with mental health professionals. In the following quotes, we mark those pastors with formal mental health training with “MHT” after their study identifier (e.g., P20, MHT).

### Theme 1: The Importance of Building Supportive Relationships

All described building supportive relationships with their congregants that included listening, advising, and counseling those in distress and their families. One pastor explained: “the role of the church is number one to comfort …I mean prayers, I mean counseling, I mean visiting, I mean encouraging” (P30, MHT). Another said their role as pastor was first to “provide love and care and counsel to these members” (P34, MHT). Supportive relationships were important: “[a person in distress] needs to have loving people around them because when you’re going through something like that …you either blame yourself, or you belittle yourself, or you reject yourself. You don’t accept yourself”(P28).

Another explained: “I always visit them at home and try…to just be friends with them, to get to know their background, what they’ve been through. Most of the time, some of them have lost a lot, and some of them were homeless… Some of them have parents at home [in Africa], and some of these things have caused so much hurt and pain… I try to find a way to know where they are coming from and get to know the root” (P32).

Once a pastor started learning more about a young person’s life, they felt able to offer more solutions, including spiritual approaches. “These people have to start telling you things about their past, their life, what they’re experiencing, and how they are trying to solve it. You are just there to help them, guide them correctly so that they will be able to be more effective. Show them other ways to solve that same problem. Show them how to pray about it” (P31).

However, to ensure that the advice would not aggravate the person’s condition, most (*n* = 12, 75%) suggested getting to know the person before engaging in activities like exorcisms of spirits. One pastor noted, “First instinct, let’s just cut out the spirit… But it may be more than that” (P23). Another offered: “Get closer to them…when you talk to them, and you realize what makes them do what they do, then you help them to change it” (P33).

The pastors identified two distinct categories of counseling: professional counseling and spiritual counseling. Professional counseling required training and focused on empowering people with mental health concerns to confront and overcome challenges and difficulties. Spiritual counseling used biblical quotations to encourage and comfort people. One pastor explained: “My wife and I are both counselors. She counsels from the side of her social work. I counsel from the Biblical side. And so, we have two approaches, but we [both] usually reach a point to at least help somebody get stable. And then we’ll refer them to a specialist” (P29).

Some pastors thought that combining professional and spiritual counseling was ideal since they thought most mental health issues were linked to demonic activities in one way or another. One said: “Pray for the person, and then do some background. Ask, ‘Are you involved in the occult?’ Some of these people, they are involved in demonic activities, and they can have these spirits …it’s the spirits that can be bothering them” (P35, MHT). If a person were having issues with demons, that would require a different approach than just counseling. This leads to our second theme, which involved identifying the source of the problem.

### Theme 2: Identifying the Source

After reading the psychosis-related vignette, most pastors described three possible causal scenarios, which were not regarded as mutually exclusive. The problem was from a physical, mental (or psychological), or spiritual source. As one explained: “[w]e do not have to limit ourselves to the spiritual dimension. We also have to look at the psychological dimension, the physical dimension” (P30, MHT).

All pastors thought the vignette represented a person with a psychological problem. Over two-thirds (*n* = 11) also described the problem in spiritual terms. About one-third also offered a physical explanation such as “brain disease.”

Many thought that spiritual and psychological distress needed to be teased apart to help a person seek out the right kind of help. Thus, they described “identifying the source” as a key part of their process that then revealed a clear course of action for effective help. A person who experienced a psychological crisis should be referred to a hospital or psychologist, for example, while a person experiencing a spiritual attack needed prayer. Many people, the pastors said, likely needed both.

To disentangle the source, many pastors asked young persons and their families for an interview. As one explained, “I do believe in science. I believe in mental health…I believe in spirituality. So, the way I come to a conclusion is to conduct an interview…And so, at an early stage, I’m able to detect…you need to go to the hospital, [or] yours is just some little spiritual thing we need to talk about it” (P34, MHT).

Pastors with clinical mental health training seemed especially concerned: “I have to be very, very careful so I don’t misdiagnose what is going on…If it is something that’s not spiritual, I can use the clinical aspect” (P35, MHT). Trained pastors used biomedical terminology, but still accentuated the importance of spiritual intervention. For example:

[J]ust sensitize the people: this is schizophrenia, it’s not a spiritual disease. Bipolar is not a spiritual disease. Depression is not a spiritual disease. All of these have spiritual dimensions to it, but these are diagnosable, empirical, socially treatable conditions that can seriously be assisted by prayers… But, just like any other disease, if it is caught earlier in the process, there’s a greater chance of solving the problem than letting it to escalate or metastasize, however you want to term it. (P30, MHT)

Untrained pastors also had a process that acknowledged medicine as relevant: “When you are praying…if the Lord directs you and says to you, ‘This is strictly spiritual,’ then follow that. This is another level. I’m sure scientists will have a different opinion… If you don’t get that guidance from the Lord telling you it’s strictly spiritual, pray, and also seek medical help” (P28).

Two-thirds (*n* = 10) of the pastors’ descriptions of the challenges congregants faced included the word “trauma.” As one explained: “We have a lot of Africans that come here with trauma. That’s why I specialize in trauma. We have gone through serious traumatic events… Some of us were brought up in the worst, crude way. Some of us were beaten like dogs. Some of us had experienced all kinds of things: hunger, disaster, poverty. Everything” (P31).

### Theme 3: Healing the Problem

Once symptoms like the ones in the vignette were clearly present, most pastors recommended that a medical professional address the problem and offer medicine while they offered support like prayer. As one explained: “If you come to me, I will tell you, ‘Look we can pray, but at the same time there is a doctor who is trained and equipped. There are hospitals that handle this kind of situation. Before we do all this prayer, please, can you at least go to the doctor and get a diagnosis, so that we can be praying while you are taking medicine’” (P30, MHT).

Most saw spiritual healing and clinical healing as complementary: “I don’t push it under the umbrella of ‘Just witchcraft.’ Period. … Just because there is a wicked spirit behind this, I don’t dismiss medical help. You know? Because we pray anyways, it’s not like we don’t pray…I think we can do both” (P23). This was important for the pastors who wanted congregants to understand that belief in demonic forces should not prevent church members from seeking medical help. One said: “I will also advise the families to seek professional help while they are also seeking spiritual help, because some people believe that this thing is purely spirit—it’s a demon from [their home] village or something like that—and sometimes it might not necessarily be demons…sometimes it’s just simple counseling, … the right words to direct to the person and then whatever-they-call-it demon would expire” (P36).

Some described praying for God to work through the doctor to identify the right diagnosis and treatment as part of the healing process:

God made the doctors, everything they had. He gave it to them—the knowledge, wisdom…that expertise can also help… So, you can go to the doctor and in your mind be praying that God will give the right medication and that medication would work…I would say, [go to] the church first, at least to give the parent or the family that peace of mind…that motivation to do it [seek medical help]. And to drive away all that shame, all that fear that you have within you, and then of course next the hospital. But I don’t want you to stay long. You definitely need both. I know God can do all things. He can heal. I know He does all things, but with both, it does help a lot (P37, MHT).

Naturally, many pastors talked about their belief in God as healer, as well as the healing power of prayer. One shared, “I’ve seen the power of God bringing the dead back to life. I’ve seen the power of God healing people” (P24). One expressed how he dealt with negative mental events in a spiritual way, interpreting such challenges as demonic: “I believe as a pastor, as a Christian, that we can attack these spirits, and we can defeat them. If we defeat the spirits, that person can feel better, so I go with that route” (P35, MHT).

Most pastors saw it as important to use both spiritual and biomedical resources to promote recovery: “I have seen some recover through the power of prayers. Others are on lifelong medication” (P27). The use of biomedicine did not contradict the power of prayer or vice-versa. “People can recover when they get the right help…both medically and spiritually” (P36). Another pastor mentioned: “We have medications that can calm those anxieties…anxiety pills. They can give them to calm them down. While they’re calming down, we can be talking to them, and praying with them and helping them” (P31). Importantly, many pastors thought recovery required both spiritual and medical resources and encouraged congregants to seek help from pastors and biomedical practitioners.

### Theme 4: Families as Partners in Care

All the pastors saw families as key to encouraging young people to seek help with mental health concerns as early as possible. One pastor described it this way:

I have this belief that you can uproot a plant, but you cannot uproot a tree. What I mean by that is that a little plant is very small; you can pull it with your finger or your hand. But a tree, you will need a lot of equipment to pull it. [So] at a very tender stage, the very moment they notice some symptoms going wrong, they should quickly seek for help. They should not pamper their children. They should seek for help…outside to see how they can rectify the case…at that young stage, they can rectify it. (P26)

Many pastors also described families as critical partners in providing care for a young person in crisis to help them move toward feeling better, as seen in the following quotation:

You see the number one need of a human being is love; to love and be loved. That’s why when people are in trouble they prefer to be surrounded by their families…it provides a certain level of comfort that nothing else would provide. That family support—that social support—medically also assists in the healing process. Just knowing that you are someone. You are loved, you are being cared for. People care about you; people care for you. I think that also helps a lot in the process. (P30, MHT)

However, pastors thought that mental health concerns were distressing for families who also needed God’s help and by proxy their help. One stated, “Every family wants to…find a way to diagnose the issue. It becomes very personal, to everyone in the house and then, they’ll take the mental issue, and make it something bigger than it’s supposed to be…God’s a healer, and they should all come together to fight the sickness rather than all of them becoming the sickness” (P32). Another described his approach: “Part of my counseling would include helping the family to have family unity and seek help…because if the family shows some love, that’s the beginning of healing” (P29). Another mentioned: “Our role is spiritual assistance. It’s standing with the family and giving the emotional support, the spiritual support they need…We bring our moral support. We stand with them” (P24).

### Theme 5: Referring and Collaborating with Mental Health Professionals

Nearly all pastors (*n* = 15, 94%) mentioned that their congregants were afraid to use doctors or hospitals for a mental health problem. Congregants, they claimed, often denied that they had a problem and refused medicine even when they were offered it (*n* = 11, 69%). Several pastors encouraged congregants to find adequate biomedical support but had mixed views on when referral to other professionals should happen in the process of supporting congregants.

Several pastors described a process in which referral came later in the process, after attempting spiritual healing or prayer. One stated, “I seek to find the root cause of their problem. I counsel them on the challenge. I pray with them. I recommend a program for them to follow. If no changes, I ask them to seek professional help” (P27). Another pastor described his process:

My first inclination will be, ‘Okay let’s take time to fast and pray, right?’… [I]f the situation is still worsening, then I will say, ‘Well, we have prayed. Not that we don’t have faith, but…the situation is still there. So, let’s then move on and see if we can set up an appointment to see a psychiatrist or psychologist, something like that.’…And don’t immediately begin injecting some medicine…You talk to them, you see what they say, and then you go from there. (P23)

Another pastor said that they had never made a mental health referral. All their church members’ “serious issues” had been resolved through spiritual healing at home. He said, “I’ve had some serious issues in the past fifteen years—very, very serious issues—and all of them we have been able to fix at their house” (P32).

In contrast, several pastors critiqued other pastors for an over-emphasis on spiritual approaches. They described pastors who sought recognition and prestige as spiritual healers, so-called “Man of God” pastors, who were less willing to make outside referrals and thus put their congregants at risk. One thought pastors had an obligation to “direct them where they need to go to get healed. It’s not a matter of saying, ‘It is my prayer that healed him.’ What matters is for the person to be healed” (P28).

Pastors who did refer to biomedical practitioners wanted to be real collaborators seen as having their own expertise. As one explained: “[S]eek our advice, too…It’s a two-way walk.” (P26). Another said: “Let’s work together because one person cannot do this. We need a team or a group of people to come. If you play your part, I play my part, [and we can] come together…to help such people” (P33).

Most pastors (*n* = 15, 94%) expressed an interest in having mental health professionals visit their churches to speak during services or offer trainings, and, as one said, “help [us] quickly identify these problems and how to recommend the right help for them” (P27). One pastor offered ideas for strengthening such relationships:

You [mental health professionals] create a relationship with the church…come around and give a talk and then…have a designated person like a chaplain [from that church] that goes to these [mental health] facilities. We have visited. Sometimes we sing our hymns with them. We pray with them. Let there be a relationship between institutions that can handle these people physically and institutions that can handle these people spiritually, and the two working together will take care of the person better. (P22)

Another pastor recommended organizing workshops “where we can have the best information on how to deal with [psychosis]. Not just focusing on our spiritual knowledge and our spiritual solutions, but also having a clear idea of what science says about it, what medicine says about it, establish a platform of collaboration that we choose. Help everybody to bring their own contribution” (P24).

## Discussion

The present study engaged sixteen African immigrant pastors in interviews to understand how pastors supported congregants with symptoms of psychosis and their families. While congregants were reluctant to seek out biomedical treatment, pastors recognized it as an important part of managing symptoms of psychosis and were eager to find more ways to collaborate with biomedical sources of support. Whether partnering with young people, families, or health professionals, all the pastors discussed the importance of relationships for care, which can be very beneficial in areas with low levels of mental health professionals. The scope of the study was limited by a small sample size in a small geographic area. Pastors in this study were well-educated and overwhelmingly male, which could have affected our results. However, the results speak to important ways forward for community mental health partnerships with pastors in providing mental health care.

Well-trained pastors can offer congregants advice, counseling, education, and follow-up support to young persons with psychosis and their families. Building close relationships with congregants experiencing mental health concerns and their families enables pastors to offer support that might prevent crises while also encouraging them to seek help from mental health professionals. While building a relationship with families is reminiscent of the “therapeutic alliance” in social work, these are typically between one person and their therapist occurring in an office setting (Castonguay et al., 2010). Thus, “therapeutic alliances” are limited by implicit and explicit rules about ethical professional boundaries and not getting too close to patients in the community (Brodwin, 2013; Floersch, 2002). However, in this case, the pastor is developing a more communal form of alliance that includes the families of the person experiencing distress and takes place in nonclinical, likely less stigmatizing settings. In addition, pastors have an established level of trust with congregants that may make it easier for them to advocate for certain kinds of care and engage families and persons with mental health concerns more effectively than others. This could be a good supplement for other kinds of social services, especially in areas with fewer resources or for cultural groups that might respond better to a trusted community member (Chaumba, 2011; Ezeobele et al., 2010; Nadeem et al., 2008; Thomas, 2008).

To do this well, African immigrant pastors (as is likely with any interested pastor) need more training, which most recognized and requested. Based on our findings, it will be important for pastors to know that in some instances (for example, intense psychotic symptoms, high substance use, intense social isolation, suicidality) delaying help seeking to get a person stable first—an approach many described trying to do through counseling and prayer throughout this paper—may not be the best plan. The American Psychiatric Association (2016) has put out a toolkit for faith-based professionals that can serve as a good resource for developing such trainings.

This study demonstrates how pastoral care may provide added, much-needed, culturally relevant support for congregants with psychotic symptoms and their families. It highlights pastors’ ability to value multiple explanatory models for their congregants, and so serve as partners in increasing congregants’ mental health literacy. The study also showed that pastors are eager to partner further with other mental health providers for the good of their congregants. Incorporating clergy into mental health care models for African immigrants may increase help-seeking, reduce treatment delays related to medical mistrust and stigma, and lead to better outcomes for young African immigrants at a higher risk of developing psychosis than their white counterparts.

## Footnotes

1 In the academic literature, religion is often associated with a specific denomination, ritual behavior, and service attendance while spirituality can be defined as connecting to a higher power through prayer or meditation (Mohr et al., 2010). However, the meanings of religion and spirituality are less distinct in the global mental health literature, which conflates terms like “faith-based,” “faith healers,” and “religious healers.” One study of Black immigrants focused on religion and spirituality also found that “religion and spirituality did not have definitive definitions in this community,” and tended to be used interchangeably.
